# Optical Coherence Tomography Angiography to Estimate Retinal Blood Flow in Eyes with Retinitis Pigmentosa

**DOI:** 10.1038/srep46396

**Published:** 2017-04-13

**Authors:** Masako Sugahara, Manabu Miyata, Kenji Ishihara, Norimoto Gotoh, Satoshi Morooka, Ken Ogino, Tomoko Hasegawa, Takako Hirashima, Munemitsu Yoshikawa, Masayuki Hata, Yuki Muraoka, Sotaro Ooto, Kenji Yamashiro, Nagahisa Yoshimura

**Affiliations:** 1 Department of Ophthalmology and Visual Sciences, Kyoto University Graduate School of Medicine, Shogoin Kawahara-cho 54, Sakyo-ku, Kyoto, Japan

## Abstract

Ophthalmologists sometimes face difficulties in identifying the origin of visual acuity (VA) loss in a retinitis pigmentosa (RP) patient, particularly before cataract surgery: cataract or the retinal disease state. Therefore, it is important to identify the significant factors correlating with VA. Nowadays, retinal blood flow in superficial and deep layers can be estimated non-invasively using optical coherence tomography angiography (OCTA). We estimated blood flow per retinal layer by using OCTA; investigated the correlation between VA and other parameters including blood flow and retinal thickness; and identified the most associated factor with VA in patients with RP. OCTA images in 68 of consecutive 110 Japanese RP patients were analysable (analysable RP group). Thirty-two age- and axial length-matched healthy eyes (control group) were studied. In the analysable RP group, the parafoveal flow density in superficial and deep layers was 47.0 ± 4.9% and 52.4 ± 5.5%, respectively, which was significantly lower than that in controls. Using multivariate analysis, we found that the parafoveal flow density in the deep layer and superficial foveal avascular area were the factors associated with VA. Non-invasive estimation of retinal blood flow per retinal layer using OCTA is useful for predicting VA in RP patients.

Retinitis pigmentosa (RP) is a major cause of visual disturbance, characterized by night blindness and visual field loss at early stage and central vision loss at advanced stage, resulting from the progressive loss of rod and cone photoreceptor cells^1^. Decreased photoreceptor density leads to visual dysfunction^2^. Retinal vasculature atrophy has been implicated in the development of RP, and anti-angiogenic therapies have been developed^3^.

Ophthalmologists sometimes face difficulties in identifying the origin of visual acuity (VA) loss in an RP patient particularly before cataract surgery: cataract or the retinal disease state. To overcome these difficulties, ophthalmologists have been seeking preoperative factors that might predict VA following cataract surgery. Yoshida *et al*. reported that the preoperative state of the ellipsoid zone, estimated using optical coherence tomography images, was an important parameter to predict the postoperative VA^4^. However retinal blood flow was not considered as a parameter.

There are some reports of blood flow evaluation in RP patients using fluorescein angiography (FA)^5^,^6^, bidirectional laser Doppler velocimetry^7^, confocal laser Doppler flowmetry^8^, magnetic resonance imaging^9^, and laser speckle flowgraphy^10^. Most of them reported that retinal blood flow of RP patients decreased. Moreover, Murakami *et al*. reported that decreased macular blood flow is associated with reduced macular visual sensitivity in RP patients^10^. However, no studies have analysed the correlation between visual function and retinal blood flow of the individual retinal layers in RP patients.

Presently, optical coherence tomography angiography (OCTA) allows for acquisition of high-resolution depth-resolved images of the chorioretinal vascular layers in a rapid, non-invasive manner without dye injection^11^. Multiple approaches for OCTA have been developed, including amplitude-based, phase-based, or combined amplitude/phase variance-based methods. One current method uses a split-spectrum amplitude-decorrelation algorithm (SSADA) that distinguishes static and non-static tissue based on the amplitude of the decorrelation coming from consecutive B-scans^12^. OCTA has been used to measure the foveal avascular zone (FAZ) area and macular vascular flow density in healthy eyes and in several diseased states^13^,^14^,^15^,^16^. Recently, a study using OCTA found that retinal blood flow density was lower in RP patients than in controls;^17^ however, the sample size was small (14 patients), and the investigators did not evaluate consecutive RP patients.

Macular oedema-free retinas are thinner in RP patients than in controls^18^. Hood *et al*. reported that the retinal nerve fibre layer of RP patients is thicker than that of controls^19^. Sandberg *et al*. showed a significant correlation between retinal thinning and lower VA in RP patients^20^. Some studies have reported that the inner segment ellipsoid band (ISe) is correlated with visual function^20^,^21^,^22^. However, the correlation between VA and retinal blood flow per retinal layer has not been investigated in RP patients.

In this study, we estimated blood flow per retinal layer by using OCTA; investigated the correlation between VA and other parameters obtained by OCTA images, including blood flow and retinal thickness; and identified the factor most associated with VA by using multivariate analysis in consecutive patients with RP.

## Results

### Demographics of the Study Population

Of the 110 RP patients, 68 and 42 were assigned to the analysable and non-analysable RP groups, respectively (Table 1). There was no significant difference in sex and axial length (AL) between analysable RP and non-analysable RP groups (*P* = 0.36 and *P* = 0.19, respectively). However, the non-analysable RP group was significantly older and had significantly lower VA than the analysable RP group (*P* = 0.02 and *P* < 0.001, respectively). It was difficult to obtain high-quality images in patients with low VA (<8/20). There was no significant difference in age, sex and AL between analysable RP and control groups (*P* = 0.25, *P* = 0.37, and *P* = 0.68, respectively). The intraclass correlation coefficient (2, 1) values for the superficial and deep FAZ area measurements were 0.99 and 0.97, respectively.

### Comparison of OCTA Image Analysis between Analysable RP and Control Groups

In the analysable RP group, the parafoveal flow density in superficial and deep layers was 47.0 ± 4.9% and 52.4 ± 5.5%, respectively, which was significantly lower than those in the control group (both, *P* < 0.001); the superficial and deep FAZ area was 0.342 ± 0.198 mm^2^ and 0.429 ± 0.154 mm^2^, respectively, which was significantly larger than in the control group (*P* = 0.03 and *P* = 0.02, respectively) (Table 2). The blood flow area of the choriocapillaris layer was not significantly different between the analysable RP and control groups (*P* = 0.34).

### Comparison of the Parafoveal Retinal Thickness and ISe Length between Analysable RP and Control Groups

In the analysable RP group, the parafoveal inner [internal limiting membrane (ILM)–inner plexiform layer (IPL)] and outer (IPL–RPE) retinal thickness was 105 ± 24 μm and 185 ± 20 μm, respectively, which was significantly thinner than those in the control group (*P* = 0.001 and *P* < 0.001, respectively); ISe length was 1687 ± 881 μm, which was significantly lower than that in the control group (*P* < 0.001) (Table 3).

### Correlation between VA and OCTA-Derived Parameters in the Analysable RP Group and between Parafoveal Flow Density and Retinal Thickness

Table 4 shows the correlation between logMAR VA and the studied parameters, including age, parafoveal flow density in superficial and deep layers, blood flow area rate of the choriocapillaris layer, superficial and deep FAZ area, parafoveal retinal thickness, and ISe length. First, using univariate analysis, we found that logMAR VA was significantly correlated with parafoveal flow density in the superficial and deep layers; superficial and deep FAZ areas; parafoveal retinal thickness of ILM–IPL and of IPL–RPE; and ISe length (*P* < 0.001, r = 0.56; *P* < 0.001, r = −0.72; *P* < 0.001, r = 0.51; *P* = 0.001, r = 0.39; *P* < 0.001, r = −0.54; *P* = 0.002, r = −0.37; *P* < 0.001, r = −0.58, respectively), but not with age and blood flow area rate of the choriocapillaris layer (*P* = 0.17 and *P* = 0.07, respectively). Second, using multivariate analysis, we found that logMAR VA was significantly correlated with parafoveal flow density in the deep layer and the superficial FAZ area (*P* < 0.001, β = −0.42; *P* = 0.003, β = −0.34, respectively). The most associated factor with logMAR VA was the parafoveal flow density in the deep layer; moreover, there was no significant correlation between logMAR VA and parafoveal flow density in the superficial layer using multivariate analysis (*P* = 0.65). Parafoveal flow density in the superficial layer was significantly correlated with parafoveal retinal thickness of ILM–IPL, but not with retinal thickness of IPL–RPE (*P* < 0.001, r = 0.51; and *P* = 0.21, respectively); parafoveal flow density in the deep layer was significantly correlated with both parafoveal retinal thickness of ILM–IPL and that of IPL–RPE (*P* < 0.001, r = 0.58; and *P* = 0.02, r = 0.27, respectively).

## Discussion

In the present study, we confirmed that the factors strongly associated with VA in RP patients were the parafoveal flow density in the deep layer and the superficial FAZ area. These parameters are significant in estimating VA in RP patients. We also found that the parafoveal flow density of retinal vessels of superficial and deep layers in RP patients was lower than that in age- and AL-matched controls. Conversely, the blood flow density of the choriocapillaris layer in RP patients was similar to that in controls. Our findings regarding the blood flow density of retinal vessels were consistent with the results of a previous study, whereas our findings for the blood flow density of the choriocapillaris layer were not^17^. However, the mean difference between RP patients and controls for blood flow density of the choriocapillaris layer in the previous report was smaller (1.9%) than that for parafoveal flow density in the superficial and deep layers (10.1% and 11.0%, respectively). Therefore, protective treatment of the retinal vessels may be effective for preventing visual loss in RP patients.

Previous reports using other investigation techniques, including bidirectional laser Doppler velocimetry, magnetic resonance imaging, and laser speckle flowgraphy reported reduced retinal blood flow^7^,^9^,^10^. In the present study, we analysed the retinal blood flow per layer, and also found that it decreased in RP patients in comparison to the contrlos. By using laser speckle flowgraphy, Murakami *et al*. reported that decreased macular blood flow is associated with reduced macular visual sensitivity in patients with RP; however, the cause-effect relationships are still to be elucidated^10^. In the present study, the parafoveal flow density in the deep layer was detected as the most associated factor with VA (*P* < 0.001, β = −0.42). It is difficult to define before cataract surgery, which is the cause of VA loss in RP patients: cataract or retinal disease state. Estimation of the parafoveal flow density in the deep layer using OCTA may help to decide if an RP patient should undergo cataract surgery.

The FAZ area of RP patients was larger than that of healthy eyes both in superficial and deep layers. Takase *et al*. reported similar results in diabetic retinopathy^23^. FAZ hardly affected the parafoveal flow density, because the parafoveal region excluded the central 0.79 mm^2^ (π × 0.5^2^) and the FAZ area was even smaller (superficial FAZ, 0.342 ± 0.198 mm;^2^ deep FAZ, 0.429 ± 0.154 mm^2^). The superficial and deep FAZ areas were significantly associated with VA using univariate analysis (*P* < 0.001 and *P* = 0.001, respectively). Furthermore, the superficial FAZ area was significantly associated with VA using multivariate analysis (*P* = 0.003, β = 0.34); however, the deep FAZ area was not associated with VA (*P* = 0.45). Therefore, the superficial FAZ area is an important factor in RP patients.

Both the parafoveal retinal thickness of ILM–IPL and that of IPL–RPE were significantly associated with VA using univariate analysis. One explanation is that in patients in advanced-stage RP, not only primary damage of photoreceptors, but also the subsequent antegrade damage of bipolar and ganglion cells may occur. Sandberg *et al*. also showed significant correlation between retinal thinning and lower visual acuity in RP patients^20^. However, other previous studies reported that the retinal nerve fibre layer is thickened in some RP patients^19^,^24^. We also observed thickened inner retina in some RP patients with better VA; however, we could not statistically analyse the relationship between thickened inner retina and VA due to the small sample size. Further research using OCTA is necessary for comparing inner retinal thickness and retinal blood flow in patients with good VA.

At the choriocapillaris level, the blood flow area rate did not differ between RP patients and controls. Two possibilities were considered. One possibility is that choriocapillaris blood flow of RP patients is not decreased even if photoreceptors, RPE, and retinal vessels are damaged. This may reflect that the primary lesion of RP is in the photoreceptors or RPE, not the choroid. Falsini *et al*. reported choroidal blood flow and choroidal velocity were reduced by 26% in RP patients compared to controls; whereas, choroidal blood volume was similar^8^. Choriocapillaris blood flow measured using OCTA indicates the blood flow volume, which is consistent with the previous report. Another possibility is the effect of artefacts. Because atrophy of the RPE in RP patients progresses with time, in the case of severe RP, permeability of OCT is increased and the blood flow image of the choriocapillaris layer can be obtained clearly, but actual choriocapillaris blood flow is decreased. Blood flow area rate obtained by OCTA may be offset. Further research is necessary to understand to which extent RPE thinning affects blood flow at the choriocapillaris level.

The limitations of this study were its cross-sectional design and the disadvantages arising from using OCTA. First, because this study was cross-sectional, RP progression was not investigated. Further longitudinal studies are necessary. Second, although OCTA has obvious advantages (e.g., it is non-invasive and allows the separate visualization of the retina and choroid compared to other instruments evaluating blood flow), OCTA has several disadvantages including the impossibility of obtaining images for cases without good fixation and endurance of blink. In 42 out of 110 RP patients (38%), we could not obtain analysable quality of OCTA images. Those patients had lower VA than the patients in the analysable RP group. Furthermore, segmentation errors may have occurred due to retinal thinning in RP patients, although we did exclude patients with cystoid macular oedema or epi-retinal membrane. Further advancements in OCTA instruments should resolve the problem.

In conclusion, quantitative OCTA analyses confirmed that the parafoveal flow density in both the superficial and deep layers decreased in RP patients compared to that in age- and AL-matched controls. The parafoveal flow density in the deep layer and superficial FAZ area are the significant factors associated with VA in RP patients. Non-invasive estimation of retinal blood flow per retinal layer using OCTA is useful for predicting VA in RP patients.

## Methods

This prospective, observational case series was approved by the ethics committee of Kyoto University Graduate School of Medicine (Kyoto, Japan). All study protocols adhered to the tenets of the Declaration of Helsinki. The nature of the study and the possible risks and benefits of participation were explained to all study candidates. All subjects choosing to participate provided written informed consent.

### Subjects

We recruited consecutive Japanese patients with RP who visited the Department of Ophthalmology and Visual Sciences at Kyoto University Graduate School of Medicine (Kyoto, Japan) between March 2016 and May 2016. All patients underwent comprehensive ophthalmological examinations, including measurement of the best-corrected VA using a decimal VA chart (Landolt chart) and AL using an IOL Master (Carl Zeiss Meditec, Inc., Dublin, CA, USA), indirect ophthalmoscopy, slit-lamp biomicroscopy, colour fundus photography and fundus autofluorescence using an Optos device (Optos PLC, Scotland, United Kingdom), and spectral domain optical coherence tomography (SD-OCT; Spectralis HRA + OCT, Heidelberg Engineering, Heidelberg, Germany). Electroretinograms were recorded according to the International Society for Clinical Electrophysiology of Vision standard protocol recommended in 2008 using LS-C (Mayo Co., Nagoya, Japan) and Neuropack MEB-2204 systems (Nihon Kohden, Tokyo, Japan). OCTA (RTVue XR Avanti with AngioVue, Optovue, Inc., Fremont, CA, USA) were also performed. Retina specialists made all diagnoses based on the above comprehensive ophthalmologic examinations. Patients with glaucoma, diabetic retinopathy, epi-retinal membrane, vitreo-macular traction syndrome, or central macular oedema were excluded. A total of 110 RP patients were included. As the control group, healthy eyes of volunteers without diabetes mellitus and hypertension, which were matched for age and AL, were enrolled.

### Obtaining Optical Coherence Tomography Angiography Images

**Figure 1:**
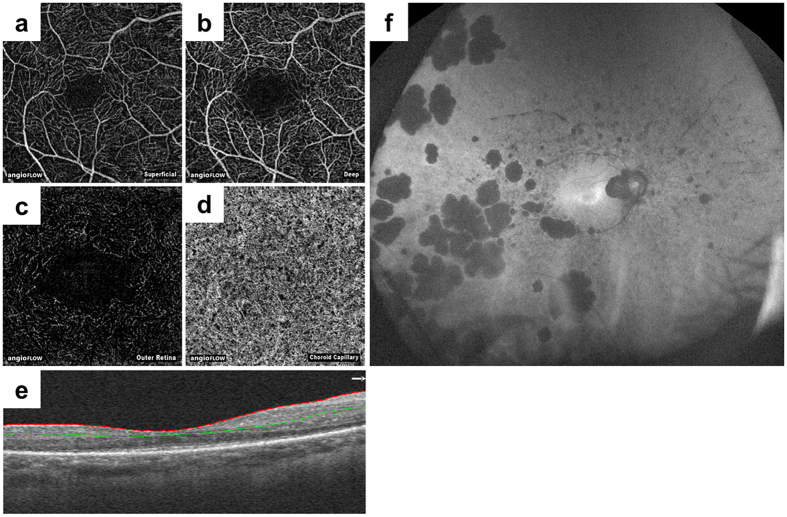
Representative optical coherence tomography angiography images and a fundus autofluorescence image. Optical coherence tomography angiography and fundus autofluorescence images of a 44-year-old woman with retinitis pigmentosa, with visual acuity of 20/16 and a mean deviation value of −12.96 dB, are depicted. The retinal layer segmentation was automatically performed by the built-in software, and created en face images of the superficial (**a**), deep (**b**), outer retinal (**c**), and choriocapillaris layers (**d**). (**e**) An optical coherence tomography image with segmentation is shown. The red line indicates the internal limiting membrane, and the green line indicates the inner plexiform layer. (**f**) A fundus autofluorescence image showing broad damage to the retinal pigment epithelium.

All OCTA images were obtained using RTVue XR Avanti with the AngioVue macular cube (3 × 3 mm) protocol. Each imaging cube comprised 304 clusters of repeated B-scans that contained 304 A-scans each. Retinal layer segmentation was automatically performed by the built-in software. The superficial capillary plexus appeared when the “en face” image was segmented between an inner boundary at 3 μm beneath ILM and an outer boundary at 15 μm beneath IPL. The en face image of the deep capillary plexus was obtained in pre-setting the inner and outer boundaries respectively at 15 μm and 70 μm beneath the IPL, corresponding to a 55 μm thick slab. Each blood flow angiography image was associated with an en face OCT image segmented at the same level and the corresponding OCT B-scan showing the level of segmentation (Fig. 1).

### Estimation of Optical Coherence Tomography Angiography Image Quality

Images obtained using OCTA were analysed using built-in software (V2015.100.0.33) based on the SSADA algorithm. First, to separate the analysable RP group and non-analysable RP group, the quality of the OCTA images for analysis were assessed by three investigators (M. S., Tomoko Hasegawa, and M. M.) who were blinded to the patient demographics, based on one or more of the following exclusion criteria reported previously^25^,^26^.

**Figure 2:**
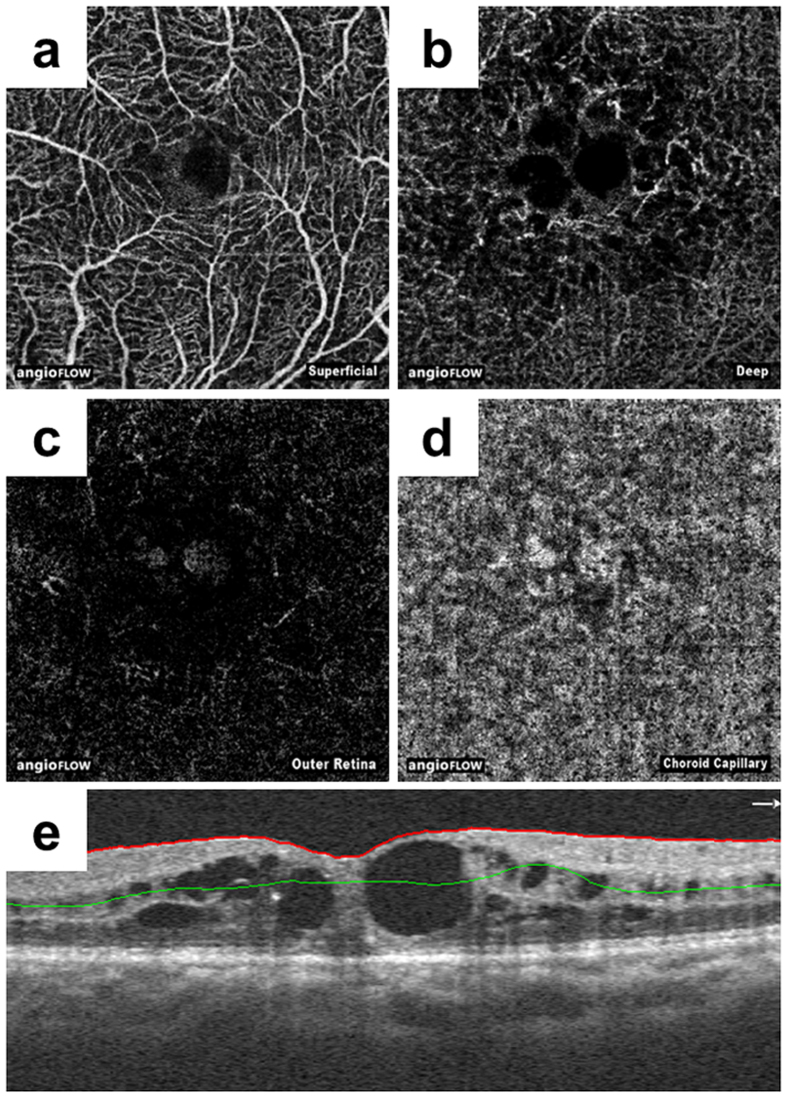
Representative optical coherence tomography angiography images of segmentation error due to cystoid macular oedema. Optical coherence tomography angiography images of a 67-year-old woman with retinitis pigmentosa with cystoid macular oedema. En face images of the superficial (**a**), deep (**b**), outer retinal (**c**), and choriocapillaris layers (**d**) are irregular due to segmentation errors (e). The red line indicates the internal limiting membrane, and the green line indicates the inner plexiform layer. We assigned these eyes to the non-analysable group.

- Low signal strength index (less than 50).
- Presence of blink artefacts.
- Poor fixation leading to motion or doubling artefacts.
- Media opacity obscuring view of the vasculature.
- Segmentation error due to cystoid macular oedema or epi-retinal membrane, among others (Fig. 2).

If OCTA images of both eyes in a patient were analysable or non-analysable, we randomly selected images of one eye for analysis. If an OCTA image of only one eye in a patient was analysable, we assigned the eye to the analysable RP group.

### Analysis of the Parafoveal Flow Density of Superficial and Deep Layers, Blood Flow Area Rate of Choriocapillaris Layer, Foveal Avascular Zone Area Retinal Thickness, and ISe Length

**Figure 3:**
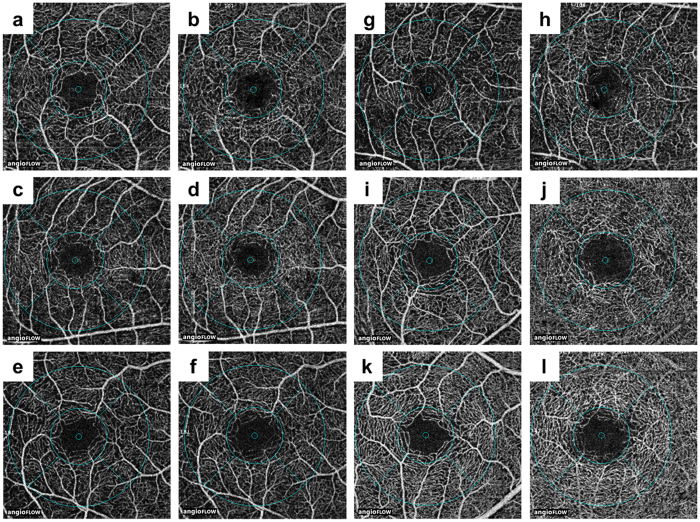
Representative optical coherence tomography images of the parafoveal flow density. Representative images of parafoveal flow density in the superficial (**a**,**c**,**e**,**g**,**i**,**k**) and deep (**b**,**d**,**f**,**h**,**i**,**j**,**l**) layers in five patients (**a**–**j**) and one control (**k**,**l**). The grid is composed of two circles centred around the fovea: an inner circle with a diameter of 1.0 mm and an outer circle with a diameter of 2.5 mm. The parafoveal region was defined as the ring between the inner and outer circles. Images of the superficial (**a**) and deep (**b**) layers of an 18-year-old female retinitis pigmentosa (RP) patient with an axial length (AL) of 23.98 mm and a parafoveal flow density of 46.94% and 53.21%, respectively. Images of the superficial (**c**) and deep (**d**) layers of a 21-year-old male RP patient with an AL of 25.10 mm and a parafoveal flow density of 46.78% and 53.83%, respectively. Images of the superficial (**e**) and deep (**f**) layers of a 44-year-old female RP patient with an AL of 24.77 mm and a parafoveal flow density of 43.66% and 51.82%, respectively. Images of the superficial (**g**) and deep (**h**) layers of a 62-year-old male RP patient with an AL of 24.55 mm and a parafoveal flow density of 41.64% and 50.88%, respectively. Images of the superficial (**i**) and deep (**j**) layers of a 62-year-old male RP patient with an AL of 23.97 mm and a parafoveal flow density of 53.85% and 59.23%, respectively. Images of the superficial (**k**) and deep (**l**) layers of a 44-year-old male control with an AL of 24.33 mm and a parafoveal flow density of 58.56% and 64.53%, respectively.

**Figure 4:**
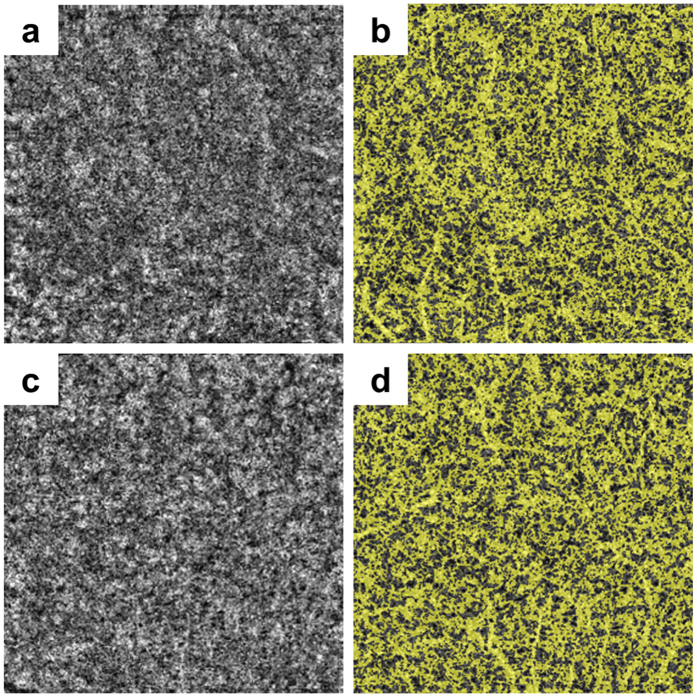
Representative optical coherence tomography images of the blood flow area rate of the choriocapillaris layer. The blood flow area rate (%) of the choriocapillaris layer was measured in the full 3.0 × 3.0 mm square using built-in software. Shown are representative images before (**a**,**c**) and after (**b,d**) automatic binarization. (**a**,**b**) Images of an 18-year-old woman with retinitis pigmentosa with a blood flow area rate of the choriocapillaris layer of 61.8%. (**c**,**d**) Images of a 44-year-old male control with a blood flow area rate of the choriocapillaris layer of 62.3%.

**Figure 5:**
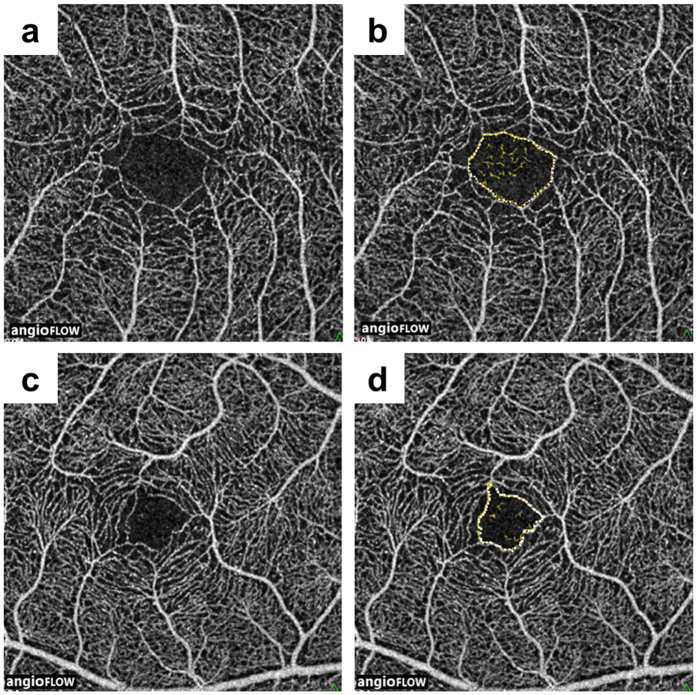
Representative optical coherence tomography images of the foveal avascular zone area. Shown are representative images of the superficial layer before (**a**,**c**) and after (**b**,**d**) processing. The foveal avascular zone (FAZ) area (mm^2^) of the superficial and deep layers was manually measured using built-in software, and the area was corrected using parameters including axial length, the flatter meridian, the steeper meridian, and the spherical equivalent refraction in Littmann’s formula. (**a**,**b**) Images of a 51-year-old woman with retinitis pigmentosa with superficial FAZ area of 0.340 mm^2^ before correction, and of 0.237 mm^2^ after correction. (**c**,**d**) Images of a 39-year-old male control with superficial FAZ area of 0.204 mm^2^ before correction, and of 0.179 mm^2^ after correction.

The software tool used a custom grid overlay that is superimposed on the 3.0 × 3.0 mm OCTA scan to generate vascular density in the different areas of interest (Fig. 3). The grid is composed of two circles centred around the fovea: an inner circle with a diameter of 1.0 mm and an outer circle with a diameter of 2.5 mm. The parafoveal region was defined as the ring between the inner and outer circles; i.e., it ranged 0.5 mm to 1.25 mm from the fovea. Flow density of retinal vasculature and retinal thickness were analysed in the parafoveal region. The blood flow area rate of the choriocapillaris layer was measured in the full 3.0 × 3.0 mm square (Fig. 4). The parafoveal flow density of superficial and deep layers and the blood flow area rate of the choriocapillaris layer were analysed by converting the obtained images to a binary format using a built-in automated thresholding algorithm. The FAZ area (mm^2^) of superficial and deep layers was manually measured using built-in software by one investigator (M.S.; Fig. 5). The area was corrected using parameters including AL, the flatter meridian, the steeper meridian, and the spherical equivalent refraction in Littmann’s formula^27^. If we could not obtain a patient’s cooperation for measuring the above parameters or we could not measure the above parameters because of a patient’s poor fixation due to low vision, we did not analyse the FAZ area of that patient. The parafoveal inner retinal thickness was automatically measured between the ILM and the IPL. The parafoveal outer retinal thickness of the parafoveal region was manually calculated by subtraction of inner retinal thickness from the automatically measured whole retinal thickness between the ILM and the retinal pigment epithelium (RPE) layer. ISe length on horizontal and vertical OCT B-scan crossing the fovea was manually measured using SD-OCT images ranged within the same flow density (within 1.25 mm from the fovea) by one investigator (M.M.). Representative measurements were used for analysis, by averaging horizontal and vertical measurements.

### Statistical Analyses

Data are presented as mean ± standard deviation where applicable. All statistical analyses were performed using SPSS (version 23, IBM, New York, USA). BCVA data were converted to the logarithm of the minimal angle of resolution (logMAR) for statistical analyses. We used *t*-tests or chi-square tests to compare data sets as appropriate. Univariate analysis was performed with Spearman’s rank correlation coefficients. Multiple stepwise regression analysis was performed with VA as the dependent variable and variables with a Spearman’s correlation with *P* < 0.10 as independent variables. The intraclass correlation coefficient values for the superficial and deep FAZ area measurements recorded by two investigators (M.S. and M.M.) in 10 randomly selected RP patients were calculated to determine the reliability of measurements. A *P*-value of <0.05 was considered statistically significant.

## Additional Information

**How to cite this article**: Sugahara, M. *et al*. Optical Coherence Tomography Angiography to Estimate Retinal Blood Flow in Eyes with Retinitis Pigmentosa. *Sci. Rep.* **7**, 46396; doi: 10.1038/srep46396 (2017).

**Publisher's note:** Springer Nature remains neutral with regard to jurisdictional claims in published maps and institutional affiliations.

## Figures and Tables

**Table 1:** Characteristics of the study population.

	RP patients			Control group
	All	Analysable RP group	Non-analysable RP group	
Eyes (patients), n	110 (110)	68 (68)	42 (42)	32 (32)
Age (years)	52.2 ± 17.8	49.9 ± 17.6	58.2 ± 16.5 (*P* = 0.02*)	54.4 ± 19.9 (*P* = 0.25)
Male sex, patients (%)	48 (46.6)	32 (47.1)	16 (38.1) (*P* = 0.36)	37.5 (*P* = 0.37)
Axial length (mm)	23.61 ± 1.39^a^	23.79 ± 1.36^b^	23.38 ± 1.46^c^(*P = *0.19)	23.89 ± 0.88 (*P = *0.68)
LogMAR VA	0.437 ± 0.685	0.160 ± 0.380	0.868 ± 0.799 (*P* < 0.001*)	−0.107 ± 0.093 (*P* < 0.001*)

Data are presented as means ± standard deviations where applicable. Analysable RP group: RP patients with analysable quality of the optical coherence tomography angiography images. Non-analysable RP group: RP patients with non-analysable quality of the optical coherence tomography angiography images. RP = retinitis pigmentosa, LogMAR VA = logarithm of minimal angle of resolution visual acuity. In ^a,b^, and ^c^, data were missing for 14, 2, and 12 eyes, respectively. *P < 0.05, t-test or chi-square test compared to the analysable RP group.

**Table 2:** Comparison of optical coherence tomography angiography images between analysable RP and control groups.

		Analysable RP group	Control group	*P*
Eyes (patients), n		68 (68)	32 (32)	
Parafoveal flow density (%)	Superficial layer	47.0 ± 4.9	55.1 ± 3.1	<0.001*
	Deep layer	52.4 ± 5.5	60.4 ± 3.1	<0.001*
Blood flow area rate in the choriocapillaris layer (%)		61.1 ± 2.8	61.5 ± 1.4	0.34
FAZ area (mm^2^)	Superficial layer	0.342 ± 0.198^a^	0.280 ± 0.083	0.03*
	Deep layer	0.429 ± 0.154^b^	0.356 ± 0.114	0.02*

Data are presented as means ± standard deviations where applicable.Analysable RP group: RP patients with analysable quality of the optical coherence tomography images.RP = retinitis pigmentosa, FAZ = foveal avascular zone.In ^a^ and ^b^, data were missing for two eyes.*P < 0.05, *t*-test.

**Table 3:** Comparison of the parafoveal retinal thickness between analysable RP and control groups.

	Analysable RP group	Control group	*P*
Eyes (patients), n	68 (68)	32 (32)	
Parafoveal retinal thickness (ILM–RPE, μm)	290 ± 41	318 ± 19	<0.001*
Parafoveal inner retinal thickness (ILM–IPL, μm)	105 ± 24	116 ± 11	0.001*
Parafoveal outer retinal thickness (IPL–RPE, μm)	185 ± 20	202 ± 11	<0.001*
ISe length (μm)	1687 ± 881	2500	<0.001*

Data are presented as means ± standard deviations where applicable.

Analysable RP group: RP patients with analysable quality of the optical coherence tomography angiography images.

RP = retinitis pigmentosa, ILM = internal limiting membrane, IPL = inner plexiform layer, ISe = inner segment ellipsoid band; RPE = retinal pigment epithelium.*P < 0.05, t-test.

**Table 4:** Correlation between visual acuity and parameters obtained using optical coherence tomography angiography in the analysable RP group (n = 68).

		Univariate analysis		Multivariate analysis	
		*P*	r	*P*	β
Age (years)		0.17	—	—	—
Parafoveal flow density (%)	Superficial	<0.001*	−0.56	0.66	—
	Deep	<0.001*	−0.72	<0.001*	−0.42
FAZ area (mm^2^)	Superficial	<0.001*^a^	0.51	0.003*	0.34
	Deep	0.001*^b^	0.39	0.45	—
Blood flow area rate of the choriocapillaris layer (%)		0.07	—	0.46	—
Parafoveal retinal thickness (μm)	Inner (ILM–IPL)	<0.001*	−0.54	0.21	—
	Outer (IPL–RPE)	0.002*	−0.37	0.09	—
Inner segment ellipsoid band length (μm)		<0.001*	−0.58	0.15	—

Analysable RP group: RP patients with analysable quality of the optical coherence tomography angiography images. FAZ = foveal avascular zone, ILM = internal limiting membrane, IPL = inner plexiform layer, RPE = retinal pigment epithelium. In ^a^ and ^b^, data were missing for two eyes. *P < 0.05, univariate analysis: Spearman’s rank correlation coefficients; multivariate analysis: multiple stepwise regression analysis.
